# Immunohistochemical expression of HER2 in breast cancer: socioeconomic impact of inaccurate tests

**DOI:** 10.1186/s12913-015-1018-6

**Published:** 2015-08-29

**Authors:** Mogens Vyberg, Søren Nielsen, Rasmus Røge, Beth Sheppard, Jim Ranger-Moore, Eric Walk, Juliane Gartemann, Ulrich-Peter Rohr, Volker Teichgräber

**Affiliations:** NordiQC, Institute of Pathology, Aalborg University Hospital, Ladegaardsgade 3, DK-9000 Aalborg, Denmark; Ventana Medical Systems Inc, 1910 E Innovation Park Drive, Tucson, AZ 85755 USA; F. Hoffmann-La Roche Ltd, Diagnostics Division, Divisional Medical and Scientific Affairs, CH-4070 Basel, Switzerland

## Abstract

**Background:**

Treatment for patients with breast cancer (BC) is guided by human epidermal growth factor receptor 2 (HER2) status. The patient’s HER2 status is assessed using US Food and Drug Administration-approved *in vitro* diagnostic (IVD) immunohistochemical (IHC) tests and laboratory-developed IVD tests. We analysed HER2 testing accuracy using data from the Nordic Immunohistochemistry Quality Control (NordiQC) HER2 IHC programme; results were used in an economic BC treatment model.

**Methods:**

Data were obtained from NordiQC HER2 BC surveys performed from 2008 to 2012. False-negative (FN) and false-positive (FP) rates for approved and laboratory-developed IVDs were used to estimate direct costs, loss of survival, productivity benefit and quality-adjusted life-years. In the absence of consistent and accessible clinical and economic data from countries participating in the NordiQC programme, United States productivity data, healthcare costs and patient numbers were used as a surrogate in order to estimate the potential impact of selecting an approved or laboratory-developed IVDs.

**Results:**

In total, 1703 tests were performed. Pooled FN rates were 11 % for approved IVDs and 25 % for laboratory-developed IVDs; FP rates were 0 % and 5 %, respectively. Using these FP and FN rates in the economic model and applying them to the United States BC population, approved IVD tests would result in better clinical outcomes, i.e., better survival and fewer disease recurrences/progressions, and lower costs, i.e., total direct costs and lost productivity, versus laboratory-developed IVD tests. Every $1 saved by laboratories by using cheaper reagents could potentially result in approximately $6 additional costs to the healthcare system.

**Conclusions:**

The results of this analysis suggest that incorrect HER2 test results have far-reaching clinical and economic consequences.

## Background

Breast cancer is the most commonly diagnosed malignancy in women worldwide and the leading cause of cancer death in developed and developing countries [1]. Breast cancer accounted for an estimated 1.67 million new cancer cases worldwide and 522 000 of all cancer deaths in 2012 [2].

The management of patients with breast cancer is complex and depends on a combination of factors. These include disease stage, age and menopausal status, the oestrogen receptor (ER) and progesterone receptor (PgR) status of the tumour, its proliferative capacity and the human epidermal growth factor receptor 2 (HER2) expression status [3].

Approximately 15–20 % of women with breast cancer have tumours that overexpress HER2 [4]. HER2 positivity is associated with aggressive disease and poor prognosis [5, 6], but the development of HER2-targeted therapies in recent years has significantly improved outcomes for patients with early stage and metastatic HER2-positive disease [7–9].

Correct histopathological assessment is the key to optimal treatment selection for patients with breast cancer. Tumour classification is based on morphological features and molecular profiles. Correct identification of tumour receptor status (ER/PgR for endocrine therapy and HER2 status for targeted therapy) is a prerequisite for treatment planning. In order to qualify for HER2-targeted therapy, the patient must have a HER2-positive tumour. This can be determined by measuring HER2 protein levels using immunohistochemistry (IHC) and/or by measuring HER2 gene amplification by *in situ* hybridization (ISH). Current guidelines recommend using either IHC or ISH to assess tumour HER2 status for all patients with breast cancer. Both tests are used, in case an equivocal result is obtained with the first test [3, 10].

The reliability and accuracy of HER2 IHC assays used in clinical practice has improved since the publication and endorsement of procedural guidelines [11, 12]. Many countries now have proficiency programmes for *in vitro* diagnostic (IVD) HER2 testing to ensure the required accuracy standards are maintained. However, not all IVD test systems perform equally. Performance depends on the quality of assay reagents and the reliability of test protocols. Some IVD tests are available as industrially produced and packaged products that are validated, approved and regulated by the US Food and Drug Administration (FDA). Other IVD tests are created by the pathology laboratories conducting the test, which assemble them from individually available components (often referred to as laboratory-developed tests). When used properly, both the approved and laboratory-developed IVDs can perform equally well; conversely, both classes of IVD have the potential to fail and produce incorrect results. Approximately 67 % of HER2 tests performed by participants in the Nordic Immunohistochemistry Quality Control (NordiQC) programme were approved and validated IVD tests, while the remaining 33 % of tests were laboratory-developed IVD tests (NordiQC 2008–2012). Similar proportions (71 % approved IVD tests and 29 % laboratory-developed IVD tests) have been reported for participants in the UK National External Quality Assessment Service (NEQAS) Breast Screening programme [13]. In some countries, the proportion of tests performed using approved IVD tests may be lower. For example, in a Belgian survey of HER2 testing, only 4 of 34 laboratories used an approved IVD testing kit [14].

The techniques and technologies underlying HER2 testing are not always made clear to oncologists because samples are processed independently in pathology laboratories; however, the oncologist needs a categorical answer regarding the patient’s HER2 status. The decision to treat a patient with HER2-targeted therapy is based largely on the reported result of the IVD test, which — if incorrect—may have significant consequences for both the patient and the society.

The aim of this study was to compare the socioeconomic consequences of the accuracy of different HER2 IVD testing procedures using data from a real-world testing/proficiency programme run by the NordiQC group. In this report we describe the reliability of results obtained from approved and laboratory-developed IVD IHC tests assessed by the NordiQC group and consider the potential clinical, economic and socioeconomic impact of inaccurate HER2 test results and subsequent treatment for patients with breast cancer.

## Methods

### Data sources

HER2 IHC testing data were collected and provided by the NordiQC organisation. These data were used in an economic model of breast cancer that was developed at Ventana Medical Systems, Inc./Roche.

#### NordiQC programme

**Fig. 1:**
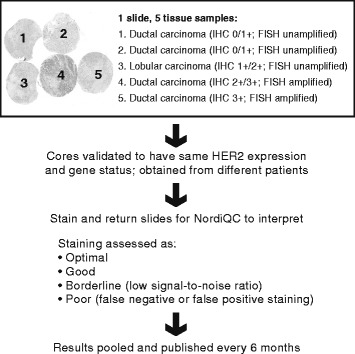
NordiQC HER2 assessment scheme. NordiQC criteria for assessing a HER2 stain as optimal are clear and unequivocal staining reactions corresponding to the 0/1+/2+/3+ IHC scores (as specified by current ASCO/CAP scoring criteria [ASCO/CAP 2007 [10]) obtained in the reference laboratories. A stain is assessed as good if a HER2-amplified 3+ carcinoma or a HER2-unamplified 0/1+ carcinoma reveals a 2+ reaction. A stain is assessed as borderline if the signal-to-noise ratio is low, for example in cases of moderate cytoplasmic reaction, excessive counterstain or excessive retrieval hampering interpretation. A stain is assessed as poor in case of a FN reaction (for example a 3+ carcinoma or a 2+ carcinoma with HER2 gene amplification that reveals a 0 or 1+ reaction) or a FP stain where a 0/1+ or 2+ carcinoma without HER2 gene amplification reveals a 3+ reaction. The inclusion of 2+ cores with and without HER2 gene amplification is considered essential to evaluate the IHC HER2 performance and the robustness of the pathology laboratories’ protocols, as 2+ cores are more likely to reveal FN or FP staining reactions than 3+ or 0/1+ cores, respectively. ASCO, American Society of Clinical Oncology; CAP, College of American Pathologists; IHC, immunohistochemistry; NordiQC; Nordic Immunohistochemistry Quality Control Group

Pathology laboratories in over 40 countries that perform IHC tests are invited to participate in a quality assessment of their immunostaining procedures as part of the NordiQC Breast Cancer HER2 IHC programme (Fig. 1). There are two shipments (or runs) per year, each of which contains a 5-core microarray slide of breast cancer cores with varying predefined HER2 expression (0/1+/2+/3+) and amplification levels (both amplified and unamplified for 2+ cancer cores) as verified by using two IHC FDA/CE-IVD approved assays (HercepTest™, Dako, Glostrup, Denmark; PATHWAY®, Ventana Medical Systems, Inc., Tucson, AZ, USA) in NordiQC reference laboratories, and using the HER2 fluorescence *in situ* hybridisation (FISH) pharmDX™ Kit (Dako). Laboratories use their own standard protocol to stain slides and return them for central assessment by the NordiQC assessor group, which is blinded to laboratory identity and assay used. False negative (FN) and false positive (FP) definitions and values obtained from the NordiQC programme were used. In brief, an FN reaction was defined as a HER2 staining reaction which was scored by the assessors as 0/1+ in a HER2 gene amplified tumour with a 2+/3+ HER2 expression in the reference laboratories. An FP reaction was defined as a HER2 staining reaction which was scored by the assessors as 3+ in a HER2 gene non-amplified tumour with a 0/1+/2+ HER2 expression in the reference laboratories.

Data from the NordiQC website for runs B6–14 were used in this analysis [15].

The study involved the use of data obtained from a quality assurance program based on anonymous human biological material collected in accordance with legislation at the site of collection; as a result, no ethics committee approvals were needed.

### Cost calculator/modelling tool

For the approved and laboratory-developed IVD tests, the possible consequences of FP and FN HER2 test outcomes were considered in relation to direct medical costs, life expectancy, quality of life and loss of productivity in patients with early stage breast cancer (EBC; stage II and III disease) and receiving systemic treatment, as well as in those with metastatic breast cancer (MBC; stage IV disease) as detailed below. When considering the differential cost of HER2 tests, reagent costs for approved and laboratory-developed IVD tests used in the NordiQC laboratories were assumed at $45 and $10, respectively [NordiQC programme, personal communication].

Survival calculations for patients with EBC were based on the results of a pooled analysis of survival among patients in the National Surgical Adjuvant Breast and Bowel Project B31 and the North Central Cancer Treatment Group trial N9831 studies [16]. For patients with MBC, survival calculations were based on the phase III H0648g study by Slamon and colleagues [7]. The number of patients who avoided disease progression because of the lower FN test rate with an approved IVD in comparison with a laboratory-developed IVD test was calculated based on disease-free survival data from the H0648g MBC trial [7].

The number of quality-adjusted life-years (QALYs) lost as a result of a patient not receiving trastuzumab was also calculated. Lost productivity was based on the loss of QALYs, assuming an interaction between QALYs and productivity, both of which are influenced by health state and deteriorate in parallel [17].

A cost calculator was developed to assess the direct financial impact of FN and FP tests. A societal perspective was adopted and US costs were used because of the homogeneity of pricing in the US healthcare system compared with the variety of systems in place in the EU. Costs were adjusted to 2014 values using an annual inflation rate of 3 % [18]. For patients with EBC, a 5-year time horizon was used to generate an annualised 1-year time horizon; a 1-year time horizon was used for patients with MBC.

All results were extrapolated to the estimated numbers of patients receiving systemic treatment for breast cancer in the US to correspond with the use of US economic data and weighted according to the observed prevalence of patients with IHC 0/1+/2+ unamplified carcinomas, 2+ amplified carcinomas and 3+ carcinomas (80 %, 4 % and 16 %, respectively) [19]. This extrapolation was performed to gain an effect of the potential costs associated with selection of the IVD test and was not intended to suggest that such costs might be saved or accrued in the US.

## Results

**Fig. 2:**
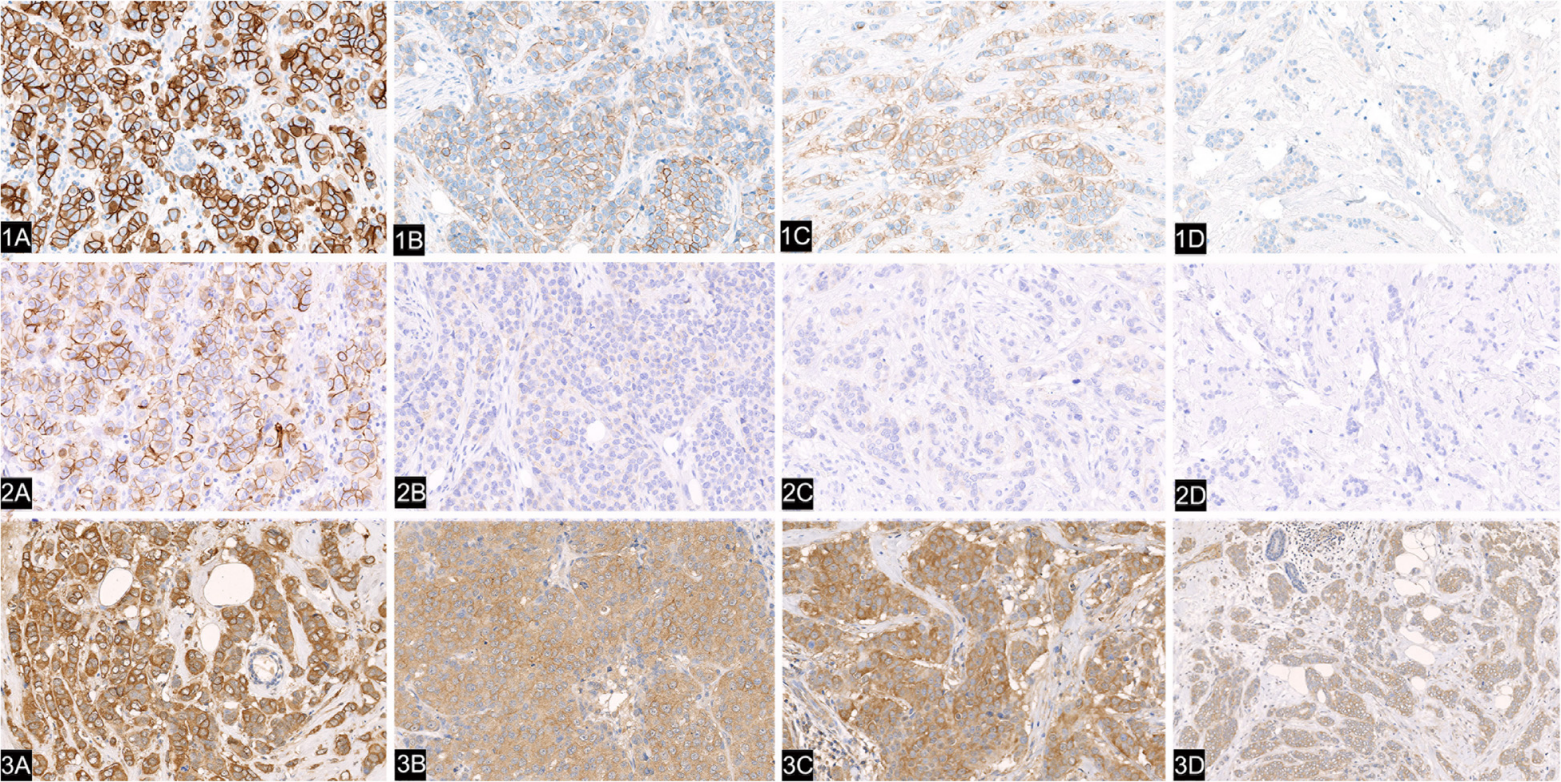
Staining reactions submitted to NordiQC based on optimal and insufficient protocols. HER2 stains of four breast carcinomas (*a*–*d*) in three laboratories (1–3). The stains from laboratory 1 (upper row) were assessed as optimal, while stains from laboratory 2 (middle row) were too weak, and from laboratory 3 (lower row) were over-stained; both sets were assessed as poor. 1*a*) In tumour *a*, >30 % of the neoplastic cells show an expected strong and complete membranous staining, corresponding to score 3+; FISH test showed amplification, HER2/CEN17 ratio >6. 1*b*) In tumour *b*, >10 % of neoplastic cells show an expected moderate complete membranous staining corresponding to score 2+; tumour *b* is amplified, HER2/CEN17 ratio 2.4–2.9. 1*c*) Tumour *c* stains as tumour *b*, but is unamplified, HER2/CEN17 ratio 1.3–1.6; 1*d*) In tumour *d*, no staining is identified; the tumour is unamplified, HER2/CEN17 ratio 1.1–1.4. 2*a*) Tumour *a* shows a 2+ reaction that would be reflexed to FISH test; 2*b*-*d*) Tumours *b*, *c* and *d* show a 0/1+ reaction. As the amplified tumour *b* would not be reflexed to FISH test, the patient would erroneously not be offered trastuzumab treatment. 3*a-d*) Widespread cytoplasmic reaction is seen in all four tumours. The stain of unamplified tumour *c* might be interpreted as a 3+; the tumour therefore would not be not reflexed to FISH test and the patient would erroneously be offered trastuzumab treatment. FISH, fluorescence *in situ* hybridisation

Key features of the NordiQC HER2 testing programme are shown in Fig. 1. Sample test results are shown in Fig. 2, highlighting accurate and inaccurate staining of the samples.

### IHC test results

NordiQC HER2 testing data obtained from runs B6 to B14 were pooled [15]. The overall FP rate was 0 % (0 of 1145 samples) for the approved IVD tests and 5 % (28 of 558 samples) for the laboratory-developed IVD tests. FN rates were 11 % (127 of 1145 samples) for the approved IVD tests and 25 % (141 of 558 samples) for the laboratory-developed IVD tests (Table 1).

**Table 1:** FP and FN rates for immunohistochemical testing as recorded by the NordiQC programme

	Approved IVD, n (%) (n = 1145)		Laboratory-developed IVD, n (%) (n = 558)	
Data source	FN	FP	FN	FP
NordiQC runs B6–14^a^	127 (11)	0	141 (25)	28 (5)

*FN* false negative, *FP* false positive, *IVD in vitro* diagnostic, *NordiQC* Nordic Immunohistochemistry Quality Control Group

^a^NordiQC IHC quality-control organisation, B6–14 runs [15]. Laboratories were provided with samples that were IHC 0, 1+, 2+ and 3+

### Impact of inaccurate results on patient outcomes

Calculations for patient outcomes were based on the estimated 232 340 patients with invasive breast cancer in the USA in 2013 [20], 132 433 (57 %) of whom are estimated to have EBC requiring systemic therapy and 16 263 (7 %) of whom have MBC; the remainder have EBC that does not require systemic therapy.

**Fig. 3:**
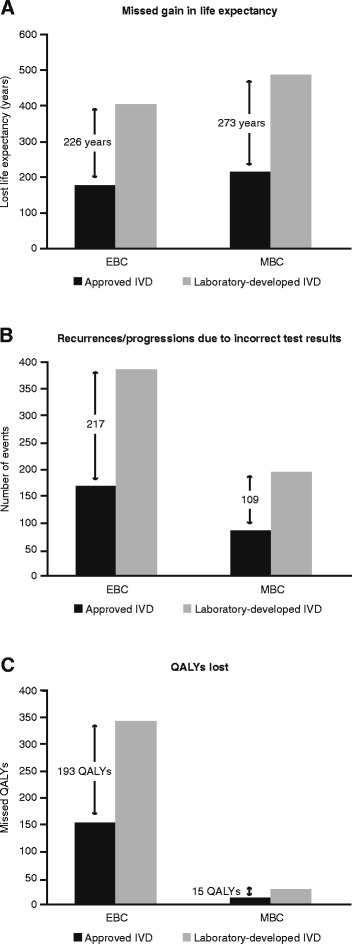
Impact of HER2 testing. **a** Missed gain in life expectancy; **b** avoidable recurrences and progressions; and **c** QALYs missed in patients with early and metastatic breast cancer based on US patient numbers and NordiQC FN and FP results; weighted according to the estimated prevalence of IHC 2+ cases (20 %). EBC, early breast cancer; FN, false negative; FP, false positive; IHC, immunohistochemistry; IVD, *in vitro* diagnostic; MBC, metastatic breast cancer; QALY, quality-adjusted life-year

For patients with EBC, an FN HER2 test was calculated to result in a difference of 3.9 % per annum in additional life expectancy that might have been achieved if the patient had received trastuzumab, based on cumulative progression over 5 years in 18.2 % of patients with EBC [16]. Using the NordiQC FP and FN rates, the estimated loss of survival per patient would be 0.0045 years for the approved IVD tests and 0.0102 years for the laboratory-developed IVD tests. Extrapolation to the US breast cancer population over a 1-year time horizon would give a total missed gain in life expectancy of 177 and 403 years, respectively, for the approved and laboratory-developed IVD tests, representing a difference of 226 years (Fig. 3a). A similar calculation was performed for patients with MBC, based on the 4.8 months’ additional life expectancy provided by trastuzumab (median overall survival 25.1 months for chemotherapy + trastuzumab vs 20.3 months for chemotherapy alone) [7]. The estimated total missed gain in life expectancy would be 215 years for the approved IVD tests and 488 years for the laboratory-developed IVD tests, representing a potential difference of 273 years for patients with MBC (Fig. 3a).

The estimated effect of an FN HER2 test on the number of patients with disease recurrence or progression was also calculated. Based on the estimated incidence of disease recurrence in 3.9 % of patients with EBC [16], 170 EBC patients tested with an approved IVD HER2 test and 387 tested with a laboratory-developed IVD HER2 test would have recurrent disease as a result of the FN test result. In total, an estimated 217 recurrences in patients with EBC could be avoided annually through the use of approved versus laboratory-developed IVD HER2 tests (Fig. 3b). In patients with MBC, based on an estimated difference in progression-free survival after 1 year of 16 % between patients treated with trastuzumab and those who did not receive trastuzumab [7], 86 patients tested with an approved IVD and 195 tested with a laboratory-developed IVD HER2 test would have progressive disease as a result of the FN result. This is the equivalent of 109 progressions in patients with MBC that could be avoided each year by using an approved instead of a laboratory-developed IVD test.

In patients with EBC, false test results would result in a missed gain in QALYs of 0.0038 for patients tested with an approved IVD compared with 0.0086 QALYs for those tested with a laboratory-developed IVD. When extrapolated to the US population, assuming a time horizon of 1 year and weighting according to the prevalence of IHC categories, this led to an estimated 149.7 missed QALYs in patients tested with an approved IVD, vs 342.3 missed QALYs in those tested with a laboratory-developed IVD, a difference of 192.5 missed QALYs in favour of the approved IVD test. In patients with MBC, the corresponding values would be 0.0024 QALYs and 0.0054 QALYs, resulting in a potential difference between approved IVD tests and laboratory-developed IVD tests of 15 QALYs when applied to the US breast cancer population (Fig. 3c).

### Economic cost of inaccurate results

As shown in Table 2, the estimated total direct cost of an FP or FN HER2 test for a patient with EBC that could be avoided each year by using an approved versus a laboratory-developed IVD HER2 test was $364 for the approved IVD tests and $1394 for the laboratory-developed IVD tests. When extrapolated to the US population with EBC and weighted according to the prevalence of the IHC categories to gain an estimate of the potential effect of use of an approved or a laboratory-developed IVD, this translated into potential total direct costs of $14 447 666 for approved IVD tests and $55 1377 720 for the laboratory-developed IVD tests. Corresponding values for MBC were $859 446 and $5 992 471, respectively (Table 3). Use of an approved IVD rather than a laboratory-developed IVD test would result in potential savings of $40 930 054 for EBC and $5 133 025 for MBC. Therefore, use of approved rather than laboratory-developed IVD tests could result in a saving of approximately $46 million.

**Table 2:** Early stage breast cancer: economic costs of inaccurate HER2 immunohistochemical test results. Based on FN and FP test results (Nordic Immunohistochemistry Quality Control Group data), US treatment costs, US patient numbers and US productivity data; weighted according to the estimated prevalence of immunohistochemistry scores

	Early stage breast cancer		
Cost, US $	Approved IVD	Laboratory-developed IVD	Difference
Direct cost/patient	364	1394	1030
Total direct costs^a^	14 447 666	55 377 720	40 930 054
Cost of lost productivity/patient^b^	83	190	107
Total cost of lost productivity^c^	3 301 263	7 546 231	4 244 968

*IVD in vitro* diagnostic

^a^Costs corrected for the prevalence of IHC 2+ tumours [19]. ^b^Productivity loss per year per patient undergoing a HER2 test. ^c^Cost for the annual number of new patients undergoing a HER2 test and receiving systemic treatment in the USA

**Table 3:** Metastatic breast cancer: economic cost of inaccurate HER2 immunohistochemical test results. Based on FN and FP test results (Nordic Immunohistochemistry Quality Control Group data), US treatment costs, US patient numbers and US productivity data; weighted according to the estimated prevalence of immunohistochemistry

	Metastatic breast cancer		
Outcome, US $	Approved IVD	Laboratory-developed IVD	Difference
Direct cost/patient	176	1228	1052
Total direct costs^a^	859 446	5 992 471	5 133 025
Cost of lost productivity/patient^b^	52	120	68
Total cost of lost productivity^c^	255 594	586 221	330 627

*IVD in vitro* diagnostic

^a^Costs corrected for the prevalence of IHC 2+ tumours [19]. ^b^Productivity loss per year per patient undergoing a HER2 test. ^c^Cost for the annual number of new patients undergoing a HER2 test and receiving systemic treatment in the USA

In terms of lost productivity, FN and FP HER2 test results associated with approved versus laboratory-developed IVD tests would result in a reduction in lost productivity estimated at $4 244 968 in patients with EBC, assuming a time horizon of 5 years, annualised to give a 1-year time horizon. In patients with MBC, the difference between approved and laboratory-developed IVD tests would be $330 627.

Despite the more expensive initial outlay for the approved rather than for laboratory-developed IVD tests, the approved IVD tests could still potentially generate considerable savings. Considering a differential cost, in terms of reagents alone, of approximately $35 between the approved and laboratory-developed IVD tests used in the NordiQC laboratories, reagents for the primary testing of US breast cancer patients with an approved IVD could potentially cost approximately $10 million per annum, with a further $15 million associated with total additional direct costs resulting from FN and FP test results. In contrast, reagent costs for primary testing with laboratory-developed IVD tests would potentially result in only approximately $2.5 million per annum in testing costs, but total additional direct costs resulting from FN and FP test results could amount to $60 million. The ratio of reagent:direct costs for the laboratory-developed IVDs ($62.5 million) and approved IVDs ($25 million) is therefore estimated at 2.5:1.

## Discussion

HER2 expression in breast cancer tissue is indicative of an aggressive pathology. HER2 expression is therefore considered a marker of poor prognosis and results from clinical trials have demonstrated a significant benefit for HER2-targeted therapy in patients with HER2-positive EBC [8] and MBC [7]. Therapeutic inhibition of the HER2 pathway has the potential to counteract the prognostic risk associated with HER2 positivity. Currently, four HER2-directed agents are available for use in the treatment of HER2-positive breast cancer: the antibodies trastuzumab, pertuzumab and trastuzumab emtansine, and the tyrosine kinase inhibitor lapatinib. However, the benefit of HER2-targeted therapies is restricted to patients with HER2 gene amplification or protein overexpression. All HER2-directed therapies can cause severe adverse events and have a heightened risk of causing significant patient harm when used in error. Therefore, they must only be administered to eligible patients most likely to respond. HER2-targeting agents are expensive medicines and are only cost-effective if used correctly. Therefore, accurate HER2 testing and HER2 status determination is fundamental to the success, safety and cost-effectiveness of breast cancer treatment programmes.

Analysis of HER2 status may be requested by oncologists who may have limited knowledge of the reliability and accuracy of the currently available HER2 testing methodologies used in the pathology laboratory. The oncologist must rely on the pathology report as the basis for selecting subsequent therapy for the patient, whose treatment outcome and safety ultimately depend on the result of this test.

Public regulations and controls on the accuracy of HER2 IVD tests are not uniformly available or executed. HER2 immunostaining must be carefully calibrated in order to define with a high degree of reliability which carcinomas are HER2 amplified. The NordiQC quality assessment programme for HER2 is based on a composition of five samples with HER2 expression of 0 to 3+ as defined in reference laboratories. Two of the samples are IHC equivocal (2+), one amplified and one unamplified, the staining reactions of which are particularly important for correct calibration. All laboratories examine the same five highly and robustly characterised tumour specimens using their own standard procedures, providing NordiQC with a good understanding of the quality of their staining procedure. When a HER2-amplified tumour with an expected 2+ IHC reaction is under-stained, giving a 1+ reaction, the tumour will be considered unamplified and not reflexed to FISH. Likewise, when a HER2-unamplified tumour with an expected 2+ IHC reaction is over-stained, giving a 3+ reaction, the tumour will be considered amplified and in most cases not reflexed to FISH. Thus, the inclusion of IHC-equivocal tumours is essential to reveal insufficient staining reactions, which may not be identified if only tumours with 0 and 3+ HER2 expression are included, as is the case in other programmes. As there is a degree of flux in participating laboratories and some additional variation in assays used even by long-term participants, there is inevitably some variation in results over time [15]. Nine consecutive runs, B6–B14, were chosen as they were based on the same composition of tumours. Run B6 was the first to include two IHC-ambiguous (2+) cancers, one being amplified and one unamplified; run B14 was the last before the ASCO/CAP guidelines were changed. As a result of the revisions to the guidelines, some tumours previously classified as 1+ are now classified as 2+, which reduced the proportion of FN cases while increasing the load of FISH tests. Examination of this series of runs, all of which were performed under consistent conditions and in line with the same guidelines, has allowed us to gain the greatest insight into and most reliable assessment of the quality of HER2 IHC testing among laboratories participating in the NordiQC programme.

In Europe, besides a general quality/safety label for medical devices, no further discrimination of HER2 test systems exists. In the USA, however, the FDA strictly oversees IVD tests that are used for any treatment decisions. For a variety of reasons, not least of which is the high development cost of such an approved test that involves extensive independent validation and proof of clinical utility from clinical trials, few diagnostic tests have been approved by the FDA. Approved IVD tests sell at a higher price than laboratory-developed IVD tests. Many laboratories are urged to be as cost-saving as possible and prefer to run laboratory-developed test systems because of the lower reagent costs involved. Our analysis was undertaken to raise awareness of the potential clinical, economic and socioeconomic differences between FDA-approved and other available assays and to provide oncologists with the information necessary to discuss with pathologists which tests to use for maximum patient benefit and safety. Moreover, it is important to demonstrate to payers that an accurate HER2 result has a greater impact on costs than simply the upfront cost of the test.

To the best of our knowledge, this is the first analysis to calculate FN and FP rates for approved and laboratory-developed IVD tests using ‘real-world’ HER2 results from a proficiency testing programme. Analysis of the NordiQC test programme provided FN and FP rates of 25 % and 5 %, respectively, for the laboratory-developed IVD tests and 11 % and 0 %, respectively, for the approved IVD tests. The large majority of inaccurate test results resulted from the failure to correctly identify IHC 2+ samples. Notably, almost all (83 %) of these incorrect results falsely identified samples as being HER2 negative, which could result in a HER2-positive patient (positivity to be confirmed by reflex ISH to determine HER2 amplification status) not receiving anti-HER2 treatment. Our findings are supported by similar results obtained by the UK NEQAS IHC Breast Screening programme: based on runs 100, 101 and 102, laboratories using approved IVD kits had pass rates of 91 %, 88 % and 94 %, compared with pass rates of 23 %, 47 % and 43 % for laboratory-developed IVD tests [13]. In a German ring study of breast cancer testing procedures, discordant results with a high percentage of FN scorings were encountered in HER2 equivocal (IHC 2+) cases compared with IHC 0/1+ and 3+ cases, with only 41 % of participants scoring these cases correctly [21]. This underlines the importance of including 2+ cases in the HER2 challenges.

Based on the economic model, which used US epidemiological and economic data in the absence of consistent, publicly available data for the countries participating in the NordiQC programme, using approved rather than laboratory-developed IVD HER2 tests could result in a saving of $46 million per year, largely as a consequence of the correct use of trastuzumab leading to avoidance of treatment costs associated with disease recurrence and progression. Although reagent costs are lower for the laboratory-developed IVD tests, the approved IVD tests are still cost saving when a broader perspective is taken, as the overall cost of using a laboratory-developed system is approximately 2.5 times greater than the overall cost of approved IVD tests. For each $1 saved by the pathology laboratory by using cheaper reagents, the healthcare system is potentially burdened with approximately $6 in additional costs. Extrapolation of these results to the EU breast cancer population, where the numbers of patients with EBC and MBC are greater than the numbers used in our analysis, suggests that the potential for savings is even greater, particularly if other HER2-overexpressing cancers are considered [22].

Some potential limitations of this analysis should be considered. The NordiQC programme uses only five tissue samples. If any of these are unusual in a way that affects the performance of approved and laboratory-developed assays differentially, this could have created bias in the comparison. We can think of no plausible mechanism by which such an interaction between sample and assay type could occur, but acknowledge that both the performance characteristics of the various assays, as well as the properties of the samples being tested, may influence the results obtained in a small testing population. In addition, samples that were most often insufficiently stained by participating laboratories are less prevalent in the general population (two of five samples [40 %] in the NordiQC array were IHC 2+, compared with a prevalence of such tumour types in the population of approximately 12 %). We allowed for this by weighting the results accordingly [19]. In addition, our calculations are based on comparing hypothetical situations in which laboratories either all use approved IVD tests or all use laboratory-developed IVD tests, whereas the reality is that a relatively small proportion of participating laboratories use laboratory-developed IVD tests. The proportion using laboratory-developed IVD tests may be significantly higher in laboratories not participating in proficiency programmes, however. Finally, this analysis was based on an economic analysis with the assumptions and estimations inherent in such models. Our intention was to provide an estimate of the potential costs of using laboratory-developed IVD tests and our results should therefore be considered as being indicative rather than absolute.

## Conclusions

The results of the present study demonstrate that the accuracy of HER2 testing has far-reaching economic, socioeconomic and clinical consequences that need to be considered when a test is requested. Oncologists should be aware that, although HER2-testing methodologies are now numerous, significant differences exist between the various available tests, which may impact on patient safety as well as outcomes. As demonstrated by the NordiQC experience [15], both the approved and laboratory-developed IVD tests can perform well and both can fail; nonetheless, the degree of regulation applied to the approved IVDs reduces the risk of failure with these agents. Adherence to testing guidelines would be expected to reassure the oncologist that accurate results can be obtained and that patients are subsequently treated correctly and not subjected to the risks associated with inappropriate therapy.

We propose that diagnostic tests impacting directly on treatment decisions, ie companion diagnostics, should be subject to in-depth regulatory scrutiny in order to ensure that all patients receive appropriate treatment. It is equally important that treatments are not incorrectly prescribed, as many recently developed agents can be very costly and may have undesired effects if used to treat the wrong patients. It is vital that the accuracy and reliability of companion diagnostic tests be maximised and that the cut-offs are validated, ideally in clinical trials or at least in prespecified analyses of retrospective samples from prospectively conducted clinical trials. Only with this level of scrutiny will patients receive the appropriate treatment and benefit from the treatment advances made in recent years.

## Abbreviations

EBC: Early stage breast cancer
ER: Oestrogen receptor
FISH: Fluorescence *in situ* hybridisation
FN: False negative
FP: False positive
FDA: Food and Drug Administration
HER2: Human epidermal growth factor receptor 2
IHC: Immunohistochemistry
ISH: *In situ* hybridization
IVD: *In vitro* diagnostic
MBC: Metastatic breast cancer
NEQAS: National External Quality Assessment Service
NordiQC: Nordic Immunohistochemistry Quality Control
PgR: Progesterone receptor
QALY: Quality-adjusted life-year
